# Coverage of fluoride data in water supply systems using the R software

**DOI:** 10.11606/s1518-8787.2022056003709

**Published:** 2022-03-29

**Authors:** Camila de Moraes Paulino, Lorrayne Belotti, Moises Kim Zanotto de Azevedo, Paulo Frazão

**Affiliations:** I Universidade de São Paulo Faculdade de Saúde Pública Departamento de Política, Gestão e Saúde São Paulo SP Brasil Universidade de São Paulo. Faculdade de Saúde Pública. Departamento de Política, Gestão e Saúde. São Paulo, SP, Brasil

**Keywords:** Water Supply, Fluoridation, standards, Water Supply Coverage, Water Quality, Big Data, Decision Making, Organizational

## Abstract

**OBJECTIVE:**

To present a protocol to criticize data on fluoride monitoring in water with R software programming features, illustrating its application to describe data coverage, and fluoridation quality in 2015.

**METHODS:**

The study used big data from the *Sistema de Informação de Vigilância da Qualidade da Água para Consumo Humano* (Information System for Surveillance of Water Quality for Human Consumption) that included all the Brazilian municipalities. Data criticism procedures were performed with the aid of R software. Filters were applied to remove municipalities with less than four months of records (1), and records with null values (2) and outliers (3). Municipalities were classified regarding the presence of valid information and fluoridation quality according to macro-region, federation units, and population size, presenting the roadmap at each step.

**RESULTS:**

Approximately 134,000 records were reviewed. Of the Brazilian municipalities, 39% had data on the fluoride parameter, and only 33.3% had four months or more of information frequency. After applying filters, 1,810 (32.5%) municipalities had valid information for the fluoride parameter, with substantial variation between the South (83.6%) and North (0.7%) macro-regions. Of these, 726 (40.1%) showed very good fluoridation quality, determined by 80% or more records within the optimal concentration interval for prevention of dental caries, with higher value (54.3%) in municipalities with 50,000 inhabitants or more, and lower (34.2%) in those with less than 10,000 inhabitants.

**CONCLUSIONS:**

Important differences persist within and between the Brazilian macro-regions regarding both the availability of information on the parameter, and the quality of water fluoridation in public supply systems in Brazil. The protocol for data review and processing with R software programming resources proved to be very useful for the production of information for decision-making based on a standardized method.

## INTRODUCTION

Water is essential for human life. Among the parameters for control of quality of water for human consumption, fluoride stands out as a health risk or protection factor, depending on its concentration. Moreover, adjusting its concentration for the purpose of preventing dental caries at a population level is recognized as a safe and effective public health intervention technology^1^. The main strategy to ensure that control is the water surveillance through an articulated system of actions that ensure data collection, analysis, and interpretation, including the rapid dissemination of results to those responsible for prevention and control^2^. The monitoring of population exposure to fluoride in water is internationally recognized as an important requirement for well-structured oral health surveillance systems^3^. Despite technological advances in fluoride concentration adjustment systems, a high variation has been found in different water supply systems in different countries^4^.

In Brazil, water surveillance activities are structured under the umbrella of the *Programa Nacional de Vigilância da Qualidade da Água para Consumo Humano* (Vigiagua – National Program for Quality Surveillance of Water for Human Consumption), supported by the Brazilian Unified Health System (SUS), and updated in 2005. Since the issuance of the Ministry of Health (MoH) Ordinance N° 1,469, of December 29, 2001, the monitoring of water quality is the responsibility of municipal health authorities, which must deploy a sampling plan, and collect samples from it. The insertion of laboratory data and the validation of the information on fluoride levels in the *Sistema de Vigilância da Qualidade da Água para Consumo Humano* (Sisagua – System for Surveillance of Water Quality for Human Consumption) is part of the set of competencies of the Federal Government, states, municipalities, and the Federal District regarding the compliance with water potability standards^8^.

Reviewing data recorded on the fluoride parameter offers relevant elements suggesting the degree of implementation of specific surveillance practices in the scope of local health organizations. The only study covering all municipalities reviewed the coverage of records for the year 2008 and showed that, through direct observation of water samples from the distribution network, fluoride surveillance was implemented in only one-third of Brazilian municipalities^9^.

The processing and interpretation of data collected by surveillance is an essential step to unveil critical points to improve public policy. Generally speaking, the time required for the analysis of a large volume of data is long if it is not performed by a specific digital resource anchored in a standardized method. The appropriation of new technological tools for data processing has become quite common among health researchers^10^. However, this incorporation is not yet a routine in the scope of public policies surveillance. The verification of coverage and quality of information assisted by digital technologies^11^ is one of the ways to raise the accuracy of records in surveillance systems^12,13^.

An overly large data set (big data) demands changes in the traditional forms of analysis, requiring apps capable of supporting their storage and processing, as well as reducing working time. The apps offered through the R programming language have advantages such as free tools; user’s independence and flexibility; adaptability of statistical methods, ensuring the resolution of future problems, including the introduction of packages that optimize the use of RAM; and a strongly active community of researchers focused on program development – a striking feature among other data analysis software^14^. Moreover, it is important to test and disseminate the most appropriate methods and programming routines so that professionals and surveillance workers can easily handle the data, and produce useful information for management.

The development of a roadmap for the criticism of fluoride concentration data in public water supply may collaborate with the use of the data produced by surveillance services, and the production of information for decision-making anchored in a standardized method. The objective of this study is to present a protocol for data criticism using the R software programming resources. It exemplifies the R software application to describe the coverage of data recorded in 2015 on fluoride concentration in water supply systems in the Brazilian municipalities, and estimate the percentage of municipalities with very good water fluoridation.

## METHODS

The article consists of the description of a customized methodological tool for verification and processing of data on fluoride concentration in water, demonstrating its application in an ecological study covering all the Brazilian municipalities. Data for 2015 recorded by Sisagua – established to support Vigiagua – and coordinated at the federal level by the Secretariat of Health Surveillance (SVS) of the Ministry of Health (MS), were used. Data were provided by the MS upon request. In addition, data on the demographic size for the year 2015 were extracted from the portal of the *Instituto Brasileiro de Geografia e Estatística* (IBGE – Brazilian Institute of Geography and Statistics).

Municipalities were classified according to the federative unit (UF) and the macro-region to which they belong. The indicators constructed were as follows: (1) rate of municipalities that systematically fed the information system, i.e., four or more months of data records on fluoride concentration during 2015^4,9^; (2) rate of municipalities with valid information, i.e., those presenting information on fluoride after applying data cleaning filters; and, (3) municipal compliance rate, defined by the ratio of cities presenting 80% or more samples within the range concentration values of best risk-benefit combination according to a technical document approved in 2011 by experts at a seminar promoted by the Centro *Colaborador do Ministério da Saúde em Vigilância da Saúde Bucal*, maintained by the University of São Paulo (CECOL-USP – Collaborating Center of the Ministry of Health in Oral Health Surveillance), a value that expresses very good quality water fluoridation^7^.

Municipalities were classified into three population size categories (< 10 thousand, 10 to < 50 thousand, and 50 thousand and more inhabitants), to allow comparison with other studies^9,15^.

### Data Criticism Protocol

The data review procedures of Sisagua were performed assisted by the free software R. The worksheet was read and then the roadmap indicated in the supplementary material^a^ was applied. Municipalities were identified as units based on the IBGE municipality code, which eliminates the risk of error due to the large number of homonymous cities in the Brazilian territory.

In the first step, the packages required to organize the database were installed and activated. It was further organized by changing the decimal separation pattern from comma to period; checking the reading of numeric and categorical variables; and, renaming the variables to avoid spaces between words by placing an underline between the word “code” and the word “IBGE” (e.g., Code_IBGE). In addition, to allow comparison with other studies^9^, we considered only data from Brasilia regarding the *Plano Piloto*, thus excluding the other administrative regions (Figure).

**Figure:**
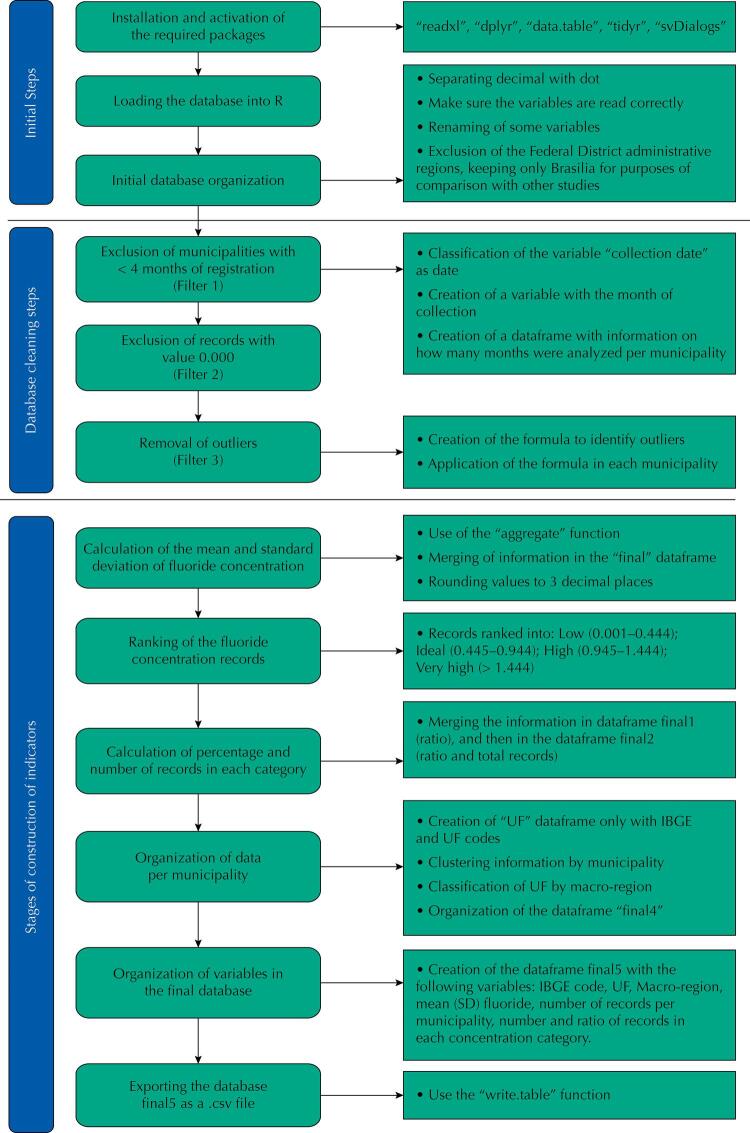
Flowchart of the database organization.

In the second stage, data were criticized using three filters applied consecutively, according to some criteria proposed by researchers^16^. The frequency of feeding the system was identified, and those municipalities with less than four months of records were excluded from the analysis (Filter 1). Records with null values were then excluded (Filter 2), and, finally, outliers in the distribution of each municipality were removed (Filter 3). Therefore, the formula for removing outliers (outliers or points outside the curve) was applied within the values of each municipality (Figure).

After each filter, an Excel spreadsheet was extracted in .xlsx format. The worksheet for Sample represents the original table with the exclusion of all municipalities with less than 4 months of information; Sample 2 represents the Sample 1 worksheet with the exclusion of reports with zero values; and, Sample 3 represents the Sample 2 worksheet with the exclusion of outliers. This extraction allowed us to identify the municipalities not included after each filter.

In the third and last step, the surveillance data on fluoride concentration in water were calculated by means of ratios and mean values per municipality. The reports of water samples, organized by municipality, were classified according to the UF and macro-region. Final data were extracted into the Final 5 spreadsheet, extension .csv, to be descriptively analyzed in Excel. In this step, information on the municipalities’ population size that remained in the database after applying the filters were also included (Figure).

To validate the procedures, data of the five cities in the Northeast showing the greatest change in relation outliers removal (Altinho (PE), Cariús (CE) Riachão do Dantas, Rosário do Catete (SE) and São Gonçalo do Amarante (RN)) were checked with the help of a calculation routine in Excel^16^.

## RESULTS

Among the 5,570 Brazilian municipalities, 39% had data on the fluoride parameter, and only 33.3% provided four months or more of information. The South (83.7%) and Southeast (36.0%) regions had the highest percentage of municipalities with four months or more of information. The Northeast (12.4%), Midwest (6.4%), and North (0.9%) regions showed the lowest percentages in this item. After applying the filters, 86.2% of the records were kept, with highest ratio in the South macro-region (90.8%), and lowest in the Midwest macro-region (65.1%), a difference of about 25 percentage points (p.p.) in the loss of records between regions (Table 1).

**Table 1:** Percentage distribution of municipalities and records after data criticism. Brazil, 2015.

	Municipalities					Post-application records				
	
	Total^a^	with Information		Filter 1^b^		Total	Filter 2^c^		Filter 3^d^	
					
	n	n	%	n	%	n	n	%	n	%
North	450	10	2.2	4	0.9	612	498	81.4	488	79.7
Roraima	15	0	0	0	-	0	0	0	0	-
Amapá	16	0	0	0	-	0	0	0	0	-
Acre	22	1	4.5	0	-	0	0	0	0	-
Amazonas	62	2	3.2	1	1.6	120	118	98.3	118	98.3
Rondônia	52	1	1.9	0	0	0	0	0	0	-
Pará	144	3	2.1	2	1.4	350	242	69.1	242	69.1
Tocantins	139	3	2.2	1	0.7	142	138	97.2	128	90.1
Northeast	1,794	352	19.6	223	12.4	15,663	11,160	71.3	10,505	67.1
Maranhão	217	10	4.6	6	2.8	439	35	8	34	7.7
Piauí	224	23	10.3	5	2.2	540	101	18.7	101	18.7
Ceará	184	175	95.1	157	85.3	10,690	8,237	77.1	7,702	72.0
Rio Grande do Norte	167	42	25.1	16	9.6	1,412	956	67.7	919	65.1
Paraíba	223	4	1.8	0	-	0	0		0	-
Pernambuco	185	9	4.9	2	1.1	46	28	60.9	19	41.3
Alagoas	102	7	6.9	1	1.0	15	15	100	12	80.0
Sergipe	75	64	85.3	28	37.3	2,114	1,595	75.4	1,540	72.8
Bahia	417	18	4.3	8	1.9	407	193	47.4	178	43.7
Midwest	466	55	11.8	30	6.4	4,020	2,749	68.4	2,617	65.1
Goiás	246	35	14.2	19	7.7	1,756	1,348	76.8	1,297	73.9
Mato Grosso do Sul	79	10	12.7	5	6.4	1,086	305	28.1	302	27.8
Mato Grosso	141	9	6.4	5	3.5	1,013	963	95.1	885	87.4
Distrito Federal^e^	1	1	-	1	100.0	165	133	80.6	133	80.6
Southeast	1,668	668	40	601	36.0	31,881	29,880	93.7	27,824	87.3
Minas Gerais	853	57	6.7	31	3.6	3,420	3,158	92.3	3,054	89.3
Espírito Santo	78	17	21.8	12	15.4	1,178	1,178	100	1,090	92.5
São Paulo	645	586	90.9	552	85.6	24,642	24,193	98.2	22,374	90.8
Rio de Janeiro	92	8	8.7	6	6.5	2,641	1,351	51.2	1,306	49.5
South	1,191	1,089	91.4	997	83.7	78,671	75,927	96.5	71,415	90.8
Paraná	399	344	86.2	304	76.2	23,521	21,118	89.8	20,212	85.9
Santa Catarina	295	260	88.1	234	79.3	10,495	10,236	97.5	9,682	92.3
Rio Grande do Sul	497	485	97.6	459	92.4	44,655	44,573	99.8	41,521	93.0
BRASIL	5,570	2,174	39.0	1,855	33.3	130,847	120,214	91.9	112,849	86.2

Source: Sisagua.

^a^ Total municipalities according to the IBGE.

^b^ Filter 1: Number of municipalities with 4 months or more of information.

^c^ Filter 2: Number of records after excluding values “0.000”.

^d^ Filter 3: Number of records after excluding outliers in the distribution by municipality.

^e^ Only data from *Brasília’s Plano Piloto* were considered, thus excluding other administrative regions of the city.

As regards records, 58.6% presented values within the concentration range considered optimal for caries prevention (0.445–0.944 mgF/L), 33.6% of the values were below this range (0.001–0.444 mgF/L), 6.5% were high values (0.945–1.444 mgF/L), and 1.3% very high values (> 1,444 mgF/L). The Southeast macro-region showed the highest percentage of records in the optimal range (88.2%), followed by the Midwest (62.9%), South (48.7%), Northeast (47.7%), and North (27.6%). In the municipalities with 50 thousand inhabitants or more, 74.4% of the records were in the optimal range, and in those with less than 10 thousand inhabitants only 42.0% were in that range, a difference of 32.4 p.p. Regarding very high values (> 1,444 mgF/L), which represent water unfit for human consumption, it is worth noting that four UF (Tocantins, Maranhão, Pernambuco, and Goiás) had 10% or more records in this situation (Table 2).

**Table 2:** Number and percentage of fluoride values in water according to concentration ranges, macro-region, federation unit, and population size. Brazil, 2015.

Macro-region	Fluoride concentration records (mgF/L)^a^								Total records

	Low (0.001–0.444)		Optimal (0.445–0.944)		High (0.945–1.444)		Very High (> 1.444)		
			
	n	%	n	%	n	%	n	%	
North	293	60.0	134	27.5	47	9.6	14	2.9	488
Roraima	0	-	0	-	0	-	0	-	0
Amapá	0	-	0	-	0	-	0	-	0
Acre	0	-	0	-	0	-	0	-	0
Amazonas	39	33.1	79	66.9	0	0.0	0	0.0	118
Rondônia	0	-	0	-	0	-	0	-	0
Pará	242	100.0	0	0.0	0	0.0	0	0.0	242
Tocantins	12	9.4	55	43.0	47	36.7	14	10.9	128
Northeast	3,914	37.3	5,008	47.7	1,370	13.0	213	2.0	10,505
Maranhão	18	52.9	12	35.3	0	0.0	4	11.8	34
Piauí	75	74.3	26	25.7	0	0.0	0	0.0	101
Ceará	2,184	28.4	4,190	54.4	1,175	15.3	153	1.9	7,702
Rio Grande do Norte	750	81.6	159	17.3	9	1.0	1	0.1	919
Paraíba	0	-	0	-	0	-	0	-	0
Pernambuco	0	0.0	0	0.0	0	0.0	19	100.0	19
Alagoas	0	0.0	10	83.3	2	16.7	0	0.0	12
Sergipe	859	55.8	528	34.3	134	8.7	19	1.2	1,540
Bahia	28	15.7	83	46.6	50	28.1	17	9.6	178
Midwest	482	18.4	1,646	62.9	124	4.7	365	14.0	2,617
Goiás	312	24.0	638	49.2	27	2.1	320	24.7	1,297
Mato Grosso do Sul	73	24.2	171	56.6	55	18.2	3	1.0	302
Mato Grosso	62	7.0	776	87.7	9	1.0	38	4.3	885
Distrito Federal^b^	35	26.3	61	45.9	33	24.8	4	3.0	133
Southeast	2,348	8.5	24,548	88.2	752	2.7	176	0.6	27,824
Minas Gerais	463	15.2	2,344	76.8	151	4.9	96	3.1	3,054
Espírito Santo	7	0.6	1,081	99.2	2	0.2	0	0.0	1,090
São Paulo	1,512	6.8	20,285	90.7	501	2.2	76	0.3	22,374
Rio de Janeiro	366	28.0	838	64.2	98	7.5	4	0.3	1,306
South	30,883	43.2	34,732	48.7	5,066	7.1	734	1.0	71,415
Paraná	4,615	22.8	14,060	69.6	1,490	7.4	47	0.2	20,212
Santa Catarina	1,430	14.8	5,173	53.4	2,676	27.6	403	4.2	9,682
Rio Grande do Sul	24,838	59.8	15,499	37.3	900	2.2	284	0.7	41,521
Population size									
Lower than 10 thousand	18,017	50.7	14,948	42.0	2,079	5.8	527	1.5	35,571
10 thousand to < 50 thousand	12,331	34.6	20,164	56.5	2,535	7.1	632	1.8	35,662
Higher or equal to 50 thousand	7,572	18.2	30,956	74.4	2,745	6.6	343	0.8	41,616
Brazil	37,920	33.6	66,068	58.6	7,359	6.5	1502	1.3	112,849

Source: Sisagua.

^a^ Results after application of Filters 1, 2 and 3.

^b^ Only data from *Brasília’s Plano Piloto* were considered, thus excluding other administrative regions of the city.

Table 3 presents the summary of this information by number and percentage of municipalities, according to the UF, macro-region, and population size. After applying Filters 2 and 3, 1,810 (32.5%) municipalities presented valid information for the fluoride parameter, of which 726 (40.1%) showed 80% or more records within the optimal concentration range (0.445–0.944 mgF/L). Important differences were observed between regions and within each macro-region. The percentage of municipalities with valid information was 83.6% in the South macro-region, with a similar pattern among its states. In the other macro-regions values were 0.7% (North), 4.7% (Midwest), 10.8% (Northeast), and 35.7% (Southeast). The states of Sergipe, Ceará, São Paulo, and the Federal District stood out with higher values in their respective regions. In the regions with highest percentage of municipalities with valid information, the quality of fluoridation measured by the percentage of municipalities that presented 80% or more of the records within the optimal concentration range was distinct. While in the South macro-region this percentage was 20.9%, in the Southeast macro-region this value was 80.4%. Values decreased as the population size decreased: 54.3% among those with 50 thousand or more inhabitants; 41.3% among those with 10 thousand or less than 50 thousand inhabitants; and 34.2% in the category with less than 10 thousand inhabitants. Considering the compliance rate over the total of municipalities, this pattern has changed in relation to states and macro-regions. According to population size, the compliance rate was virtually the same when comparing municipalities with 10 to 50 thousand inhabitants with those with less than 10 thousand inhabitants.

**Table 3:** Number and percentage of municipalities with valid information and very good fluoridation quality according to macro-region, federation unit, and population size.

	Municipalities					

	Total^a^	With valid information^b^		Compliance rate		
		
	n	n	%	n	%^c^	%^d^
North	450	3	0.7	0	-	-
Roraima	15	0	-	0	-	-
Amapá	16	0	-	0	-	-
Acre	22	0	-	0	-	-
Amazonas	62	1	1.6	0	-	-
Rondônia	52	0	-	0	-	-
Pará	144	1	0.7	0	-	-
Tocantins	139	1	0.7	0	-	-
Northeast	1,794	193	10.8	32	16.6	1.8
Maranhão	217	3	1.4	0	-	-
Piauí	224	1	0.5	0	-	-
Ceará	184	137	74.5	28	20.4	15.2
Rio Grande do Norte	167	16	9.6	0	-	-
Paraíba	223	0	-	0	-	-
Pernambuco	185	2	1.1	0	-	-
Alagoas	102	1	1.0	1	100.0	1.0
Sergipe	75	27	36.0	1	3.7	1.3
Bahia	417	6	1.4	2	33.3	0.5
Midwest	466	22	4.7	7	31.8	1.5
Goiás	246	16	6.5	5	31.3	2.0
Mato Grosso do Sul	79	2	2.5	0	-	-
Mato Grosso	141	3	2.1	2	66.7	1.4
Distrito Federal	1	1	100.0	0	-	0.0
Southeast	1,668	596	35.7	479	80.4	28.7
Minas Gerais	853	26	3.1	15	57.7	1.8
Espírito Santo	78	12	15.4	12	100.0	15.4
São Paulo	645	552	85.6	452	81.9	70.1
Rio de Janeiro	92	6	6.5	0	-	-
South	1,191	996	83.6	208	20.9	17.5
Paraná	399	304	76.2	131	43.1	32.8
Santa Catarina	295	233	79.0	35	15.0	11.9
Rio Grande do Sul	497	459	92.4	42	9.2	8.5
Population size						
Lower than 10 thousand	2,451	833	34.0	285	34.2	11.6
10 thousand to < 50 thousand	2,464	688	27.9	284	41.3	11.5
Higher or equal to 50 thousand	655	289	44.1	157	54.3	24.0
Brazil	5,570	1,810	32.5	726	40.1	13.0

Source: Sisagua.

^a^ Total municipalities according to the IBGE.

^b^ Municipalities with valid information after applying Filters 1, 2 and 3.

^c^ Percentage corresponds to the municipalities that presented 80% or more of records within the optimal concentration range (0.445–0.944) in relation to the municipalities that had valid information.

^d^ Percentage corresponds to the municipalities that presented 80% or more of records within the optimal concentration range (0.445–0.944) in relation to the total of municipalities.

In 2015, 16 capital cities and the Federal District used to fluoridate their water. Among them, 11 (64.7%) had valid records of fluoride concentration, and five (29.4%) showed 80% or more of the records within the optimal concentration range.

## DISCUSSION

The main contribution of this study was to present a roadmap for criticizing big data with fluoride concentration values in public supply water, thus showing its application to describe data recorded in 2015 in Brazil. Results showed important variations that require coordinated action among the many spheres of government responsible for managing the Vigiagua. The use of the R software programming language allowed the organization and analysis of about 134 thousand records about the fluoride parameter present in water supply systems, concerning the year 2015, distributed over different Brazilian municipalities. Summarization was important to identify differences between and within regions, both on the availability of information on the parameter, and on the quality of fluoridation of public water supply in Brazil.

The use of increasingly robust software requires equipment suitable to run dense programs and files, in addition to the costs for acquiring plans that allow access to the resource. The selection of analysis tools depends on the user’s goals, the resources most used in their professional environment, and solutions that are easily implemented and favor decision-making. In general, one should look for tools that are flexible, widely used, well documented, and robust enough to meet the intended goals. Besides its unlimited analytical capacity, R is regularly updated, has great graphical features, can be used online, and is free of charge to users. There are hundreds of packages on various servers at universities and institutes with functions, algorithms, and procedures for various types of data processing. The large community of users that adopted R mean it is less prone to errors compared to other programming languages. Aiming at expanding the use of R, research in the health area has been dedicated to describe the step-by-step analysis with this language, exemplifying functions for reading and manipulating data^14,17^, as well as creating and making available packages for its upgrading^18^.

However, although it is becoming more common among researchers, its use as a tool for data management is still limited, since it requires the constant training of professionals and data literacy to manage computational languages^19^. The roadmap presented in this manuscript can be adapted to other datasets with some modifications, being a flexible and free alternative. The allocation of resources in the budget of health management agencies and the provision of training activities are important measures to overcome the barriers between technology and surveillance and health care services.

Approximately two-thirds of the Brazilian municipalities did not have valid information for the fluoride parameter, most of them being located in the North, Northeast, and Midwest regions, having less than 50 thousand inhabitants. This situation is virtually the same as that observed in 2008, when researchers found underfeeding and absence of data on fluoride in 62.7% of the Brazilian municipalities, mainly in sites with worse socioeconomic and health indicators. They warned about problems in the structure of Sisagua and in its use by the municipalities, recommending changes in the system aimed at improving and fulfilling its purpose^9^.

Although the information system has undergone very important changes^8^, we can infer that seven years later the implementation of the national water surveillance program regarding the fluoride parameter, after experiencing an initial stage of expansion until 2008, is almost stagnant. We consider this a worrisome situation that requires action from the Brazilian health authorities, among other agencies, such as the public prosecutors’ offices^20,21^ and consumer protection and defense agencies^22^. Some UF, where the frequency of valid information is low, urges for the formulation of strategies to insert the theme into the agenda of health managers.

In 2005, of the 17 Brazilian capitals that were fluoridated, including the Federal District, only 29.4% performed the steps of collection, analysis, and disclosure of the fluoride parameter^23^. Ten years later, the ratio rose to 64.7%. A multicenter study in Brazilian municipalities with more than 50,000 inhabitants indicated that 2/3 of them were provided with water fluoridation, and 53% performed fluoride concentration surveillance based on external control data (heterocontrol data), with higher percentages in the South and Southeast regions^15^. According to this study, 44.1% of the municipalities with more than 50,000 inhabitants had valid information based on heterocontrol data. The difference may be related to the design of each study. While in that study the estimate was calculated for the period between 2010 and 2015, and included only fluoridated municipalities with population coverage above 49.9%, in this one the estimate took into account only the year 2015 and included all municipalities with valid information frequency in the system, regardless of the ratio of population covered by fluoridation.

Overcoming current limitations is essential for the monitoring and evaluation of population exposure to fluoride. The identification of areas where the degree of implementation of the surveillance program is very low may guide decision making. The results achieved with initiatives to improve both coverage and quality of the mortality system notification by the epidemiological surveillance teams at the federal, state, and municipal levels reveal the relevance of these actions^24^, which should take the form of a permanent effort to reduce regional differences in the quality of records^25^.

The low quality of information in many municipalities may be linked to difficulties related to the availability of structural resources needed to ensure proper feeding of the system, such as appropriate computers and Internet access in the work environment^26^. The challenges for structuring it, such as the reference in data registration and transparency in the disclosure of information about water supply in Brazil, also include the raising awareness of those involved regarding the need for data input in the system, and its importance for the management of health risks associated with water supply in the country^8^.

Regarding valid information, more than half of it was within the optimal concentration range. Similar to the evidence summarized by a literature review^4^, non-compliance was proportionally higher towards low values, which could mean increased caries risk, than towards values above the optimal concentration range, which could imply higher risk of dental fluorosis. In England, fluoride concentrations in areas served by public health technology were also lower than the target set at 1 mgF/L^6^. Among the factors that could cause fluctuations in concentration, the following have been highlighted: lack of fluoride equipment/substance; laboratory and technical infrastructure; technical-operational difficulty due to lack of training programs; and complexity of the distribution network^4,6^. However, huge disparities were observed between and within regions. One can assume that the information system may not be being used in a timely manner by public agents to warn those responsible for water treatment in the supply systems about required corrective actions.

Among municipalities that had valid information about the fluoride parameter, less than half showed 80% or more records in the optimal concentration range. The highest percentages were observed in the Southeast macro-region, and in the municipalities with 50 thousand inhabitants or more. A study carried out in the state of São Paulo showed that municipalities which had not reached this standard had smaller population size, lower per capita income, and the supply was not provided by the state company adjusted by other municipal indicators^7^. Research carried out in an important Brazilian metropolitan region showed that water fluoridation quality was higher, the higher the value of human development index, population size, coverage of supervised tooth brushing, and the lower the infant mortality rate and ratio of tooth extraction procedures to total basic procedures^27^.

Considering that the reduction of socioeconomic bias in dental caries distribution, as a result of the proper adjustment of fluoride concentration in water, is ensured when the water supply network reaches rich and poor neighborhoods^28^, a hypothesis for future studies would be to check whether socioeconomic conditions are worse among Brazilian municipalities that have not fulfilled their obligations regarding water surveillance on fluoride parameter. The lack of population exposure to fluoride in the water supply in territories where concentration adjustment could bring more benefits due to difficulties in access to other sources of fluoride to protect human dentition, configures a source of social injustice. It may also interfere with the cost-effectiveness of this measure, since, in addition to increasing the risk of dental caries, the costs of implementing and maintaining fluoridation would not be accompanied by the expected public health benefits.

One of the limitations of this study is to read the scope of findings. In this sense, it is important to highlight that we considered all municipalities with four or more months of registration, and that a more refined criterion, including only municipalities with six or eight months of registration, could generate different results. However, the criterion adopted enabled comparing data from seven years ago, and had as a reference the heterogeneity of the Vigiagua implementation process in Brazil. Another point to note is the need for adjustments to the original roadmap, and the creation of new filters for the evaluation of other water quality parameters, in accordance with current regulations. Accordingly, R provides different statistical tools that could complement the analysis described which, in this case, prioritized data processing according to the proposed objectives.

The analysis of quality of the data recorded for 2015 showed that two-thirds of the Brazilian municipalities did not have valid information for the fluoride parameter, suggesting that the implementation of the national water surveillance program regarding fluoride parameters has not improved since 2008. Among the municipalities that performed direct observation of water samples from the distribution network, 40.1% showed very good fluoridation quality standard, with important differences by population size, macro-region, and UF. The roadmap for criticism and handling of data with R software programming resources proved to be very useful for the production of information aimed at decision-making anchored in a standardized method.

## Footnotes

a Supplementary material available from: http://www.cecol.fsp.usp.br/dcms/uploads/arquivos/1646748853_Material%20Suplementar%20RSP%202022%20vol%2022%20Paulino%20et%20al..pdf
