# Supplementary material for: Coverage of fluoride data in water supply systems using the R software
Source: Rev Saude Publica. 2022 Mar 29;56:18. doi: 10.11606/s1518-8787.2022056003709 (PMC8973027; doi:10.11606/s1518-8787.2022056003709)
Supplement: Translation

# Translation: Cobertura dos dados de fluoreto em sistemas de abastecimento de água usando o software R

**Authors:** Camila de Moraes Paulino, Lorrayne Belotti, Moises Kim Zanotto de Azevedo, Paulo Frazão

**Affiliations:** I Brasil Universidade de São Paulo. Faculdade de Saúde Pública. Departamento de Política, Gestão e Saúde. São Paulo, SP, Brasil

**Keywords:** Abastecimento de Água, Fluoretação, normas, Qualidade da Água, Cobertura do Serviço de Água, Big Data, Tomada de Decisões Gerenciais

## Abstract

**OBJETIVO:**

Apresentar um protocolo para a crítica dos dados sobre monitoramento do fluoreto na água com recursos de programação do software R, ilustrando sua aplicação para descrever a cobertura dos dados e a qualidade da fluoretação em 2015.

**MÉTODOS:**

Foi utilizado *big data* proveniente do Sistema de Informação de Vigilância da Qualidade da Água para Consumo Humano que incluía todos os municípios brasileiros. Os procedimentos de crítica dos dados foram realizados com auxílio do software R. Aplicaram-se filtros para remover municípios com menos de quatro meses de registro (1), e registros com valores nulos (2) e aberrantes (3). Os municípios foram classificados quanto à presença de informações válidas e à qualidade da fluoretação segundo macrorregião, unidades da federação e porte populacional, apresentando-se o script em cada passo.

**RESULTADOS:**

Foram analisados aproximadamente 134 mil registros. Dos municípios brasileiros, 39% possuíam dados sobre o parâmetro fluoreto e apenas 33,3% apresentaram quatro meses ou mais de frequência de informação. Após a aplicação dos filtros, 1.810 (32,5%) municípios apresentaram informação válida para o parâmetro fluoreto, com substancial variação entre as macrorregiões Sul (83,6%) e Norte (0,7%). Destes, 726 (40,1%) exibiram muito boa qualidade da fluoretação, determinada por 80% ou mais de registros dentro do intervalo concentração ótima para prevenção da cárie dentária, com valor mais alto (54,3%) nos municípios com 50 mil habitantes ou mais e mais baixo (34,2%) naqueles com menos de 10 mil habitantes.

**CONCLUSÕES:**

Persistem importantes diferenças entre e dentro das macrorregiões brasileiras no que se refere tanto à disponibilidade da informação sobre o parâmetro quanto à qualidade da fluoretação da água nos sistemas de abastecimento público no Brasil. O protocolo para crítica e manuseio dos dados com recursos de programação do software R se mostrou bastante útil para produção de informação voltada a tomada de decisão ancorada em método padronizado.

## INTRODUÇÃO

A água é essencial para a vida humana. Entre os parâmetros para controle da qualidade da água para o consumo humano, destaca-se o fluoreto por ser, conforme sua concentração, fator de risco ou de proteção à saúde. Além disso, o ajuste da sua concentração para fins de prevenção da cárie dentária em nível populacional é reconhecido como uma tecnologia de intervenção em saúde pública segura e efetiva^1^. A principal estratégia para assegurar esse controle é a vigilância da água por meio de um sistema articulado de ações que asseguram a coleta, análise e interpretação de dados, incluindo a rápida disseminação dos resultados para todos aqueles que são responsáveis pela prevenção e controle^2^. O monitoramento da exposição populacional ao fluoreto presente na água é reconhecido internacionalmente como um importante requisito de sistemas de vigilância da saúde bucal bem estruturados^3^. No que pese o avanço tecnológico dos sistemas de ajuste da concentração do fluoreto, uma variação elevada tem sido encontrada em diferentes sistemas de abastecimento de água de diferentes países^4^.

No Brasil, as atividades de vigilância da água estão estruturadas sob a égide do Programa Nacional de Vigilância da Qualidade da Água para Consumo Humano (Vigiagua), mantido pelo Sistema Único de Saúde (SUS) e atualizado em 2005. Desde a publicação da Portaria do Ministério da Saúde (MS) n° 1.469, de 29 de dezembro de 2001, o monitoramento da qualidade da água é responsabilidade das autoridades sanitárias municipais, que devem implementar um plano de amostragem e coleta de amostras dela. A inserção dos dados laboratoriais e a validação da informação sobre os teores de fluoreto no Sistema de Informação de Vigilância da Qualidade da Água para Consumo Humano (Sisagua) é parte do conjunto de competências da União, estados, municípios e Distrito Federal voltadas ao cumprimento das normas de potabilidade da água^8^.

O exame dos dados registrados sobre o parâmetro fluoreto oferece elementos relevantes sugestivos do grau de implementação de determinadas práticas de vigilância no âmbito das organizações sanitárias de nível local. O único estudo que abrangeu todos os municípios examinou a cobertura dos registros relativos ao ano de 2008 e mostrou que, por meio da observação direta de amostras de água provenientes da rede de distribuição, a implementação da vigilância do fluoreto tinha alcançado somente um terço dos municípios brasileiros^9^.

O processamento e interpretação dos dados coletados pela vigilância é uma etapa essencial para desvelar os pontos críticos visando a melhoria da política pública. Geralmente, o tempo demandado para a análise de um grande volume de dados é longo se não executado por um recurso digital específico ancorado em método padronizado. A apropriação de novas ferramentas tecnológicas para o tratamento de dados tornou-se bastante habitual entre pesquisadores da área de saúde^10^. Entretanto, no âmbito da vigilância de políticas públicas, essa incorporação ainda não é uma rotina. A verificação da cobertura e da qualidade da informação com auxílio de tecnologias digitais^11^ representa um dos caminhos para elevar a acurácia dos registros nos sistemas de vigilância^12,13^.

Um conjunto de dados demasiadamente grande (*big data*) demanda mudanças nas formas tradicionais de análise, exigindo aplicativos capazes de suportar o armazenamento e processamento deles, além de reduzir o tempo de trabalho. Aqueles oferecidos por meio da linguagem de programação R trazem vantagens, como a gratuidade de instrumentos; independência e flexibilidade do usuário; adaptabilidade dos métodos estatísticos, assegurando a resolução de problemas futuros, inclusive, a introdução de pacotes que otimizem o uso da memória RAM; e uma comunidade fortemente ativa de pesquisadores focada no desenvolvimento do programa, característica marcante entre os demais softwares de análise de dados^14^. Ademais, é importante testar e disseminar os métodos e as rotinas de programação mais apropriadas a fim de que profissionais e trabalhadores da vigilância possam manusear os dados com facilidade e produzir as informações que são úteis para a gestão.

A elaboração de um script para a crítica de dados de concentração de fluoreto na água de abastecimento público pode colaborar com o uso dos dados produzidos pelos serviços de vigilância e a produção de informação para tomada de decisão ancorada em método padronizado. O objetivo deste estudo é apresentar um protocolo para a crítica dos dados com recursos de programação do software R, ilustrando sua aplicação para descrever a cobertura dos dados registrados em 2015 sobre concentração do fluoreto em sistemas de abastecimento de água nos municípios brasileiros e estimar a porcentagem de municípios com fluoretação da água de muito boa qualidade.

## MÉTODOS

O artigo consiste na descrição de uma ferramenta metodológica customizada para a verificação e processamento dos dados de concentração de fluoreto na água, ilustrando, assim, sua aplicação em um estudo ecológico que abrangeu todos os municípios brasileiros. Foram utilizados dados registrados para o ano de 2015 provenientes do Sisagua, criado para apoiar o Vigiagua, coordenado em nível federal pela Secretaria de Vigilância em Saúde (SVS) do Ministério da Saúde (MS). Os dados foram fornecidos pelo MS mediante solicitação. Além disso, dados sobre o porte demográfico relativos ao ano de 2015 foram extraídos do portal do Instituto Brasileiro de Geografia e Estatística (IBGE).

Os municípios foram classificados segundo a unidade federativa (UF) e a macrorregião a que pertencem. Os indicadores construídos foram: (1) taxa de municípios que alimentaram o sistema de informação de forma sistemática, ou seja, quatro ou mais meses de registros de dados de concentração de fluoreto durante o ano de 2015^4,9^; (2) taxa de municípios com informações válidas, ou seja, aqueles municípios que apresentaram informação sobre o fluoreto após a aplicação dos filtros de limpeza de dados; e (3) taxa de conformidade municipal, definida pela proporção de cidades que apresentaram 80% ou mais de amostras dentro do intervalo valores de concentração de melhor combinação risco-benefício de acordo com documento de técnico aprovado, em 2011, por especialistas em seminário promovido pelo Centro Colaborador do Ministério da Saúde em Vigilância da Saúde Bucal, mantido pela Universidade de São Paulo (CECOL-USP), valor que expressa fluoretação da água de muito boa qualidade^7^.

Os municípios foram classificados em três categorias de porte populacional (< 10 mil, 10 a < 50 mil, e 50 mil e mais habitantes), para permitir comparação com outros estudos^9,15^.

### Protocolo de Crítica dos Dados

Foram efetuados procedimentos de crítica dos dados do Sisagua com auxílio do software livre R. A planilha de trabalho foi submetida à leitura e, depois, foi aplicado o script indicado no material suplementar^a^. Os municípios foram identificados como unidades a partir do código de município do IBGE, o que suprime o risco de erro pelo grande número de cidades homônimas no território nacional.

Na primeira etapa, foram instalados e ativados os pacotes necessários para organização do banco. Depois, foi realizada a organização dele por meio da alteração do padrão de separação decimal de vírgula para ponto; checagem da leitura das variáveis numéricas e categóricas; e, renomeação das variáveis para evitar espaços entre palavras colocado um traço sublinear entre a palavra “código” e a palavra “IBGE” (ex.: Codigo_IBGE). Além disso, para permitir a comparação com outros estudos^9^, foram considerados apenas os dados de Brasília concernentes ao Plano Piloto, excluindo-se, portanto, as demais regiões administrativas (Figura).

**Figura:** Fluxograma da organização do banco de dados.

Na segunda etapa, os dados foram submetidos à crítica, utilizando-se três filtros aplicados consecutivamente, conforme alguns critérios propostos por pesquisadores^16^. Foi identificada a frequência de alimentação do sistema e, aqueles municípios com menos de quatro meses de registros, foram excluídos da análise (Filtro 1). Em seguida, foram excluídos os registros com valores nulos (Filtro 2), e, por fim, foram removidos os valores aberrantes na distribuição de cada município (Filtro 3). Portanto, a fórmula para remoção de *outliers* (valores aberrantes ou pontos fora da curva) foi aplicada dentro dos valores de cada município (Figura).

Após cada filtro, foi extraída uma planilha de Excel no formato .xlsx. Desta forma, a planilha Amostra 1 representa a tabela original com a exclusão de todos os municípios com menos de 4 meses de informação; a Amostra 2 representa a planilha Amostra 1 com a exclusão de laudos com os valores zerados; e, a planilha Amostra 3 representa a planilha Amostra 2 com a exclusão dos *outliers*. Essa extração permitiu identificar os municípios não incluídos após cada filtro.

Na terceira e última etapa, os dados de vigilância da concentração de fluoreto na água foram apurados por meio do cálculo das proporções e dos valores médios por município. Os laudos das amostras de água, organizados por município, foram classificados segundo a UF e a macrorregião. Os dados finais foram extraídos para a planilha Final 5, de extensão .csv, para serem analisados descritivamente no Excel. Nesta etapa, também foram incluídas as informações sobre o porte populacional dos municípios que permaneceram no banco após a aplicação dos filtros (Figura).

Para validar os procedimentos, os dados das cinco cidades do Nordeste com maior mudança em relação à exclusão de *outliers* (Altinho (PE), Cariús (CE) Riachão do Dantas, Rosário do Catete (SE) e São Gonçalo do Amarante (RN)) foram conferidos com auxílio de uma rotina de cálculos no Excel^16^.

## RESULTADOS

Dentre os 5.570 municípios brasileiros, 39% possuíam dados sobre o parâmetro fluoreto e apenas 33,3% apresentaram quatro meses ou mais de informação. As regiões Sul (83,7%) e Sudeste (36,0%) foram as que apresentaram o maior percentual de municípios com quatro ou mais meses de informação. As regiões Nordeste (12,4%), Centro-Oeste (6,4%) e Norte (0,9%) apresentaram os percentuais mais baixos nesse item. Após a aplicação dos filtros, mantiveram-se 86,2% dos registros, com maior proporção na macrorregião Sul (90,8%) e menor na macrorregião Centro-Oeste (65,1%), uma diferença de cerca de 25 pontos percentuais (p.p.) na perda de registros entre as regiões (Tabela 1).

**Tabela 1:** Distribuição percentual dos municípios e dos registros após a crítica dos dados. Brasil, 2015.

	Municípios					Registros após aplicação				
	
	Total^a^	Com informação		Filtro 1^b^		Total	Filtro 2^c^		Filtro 3^d^	
					
	n	n	%	n	%	n	n	%	n	%
Norte	450	10	2,2	4	0,9	612	498	81,4	488	79,7
Roraima	15	0	0	0	-	0	0	0	0	-
Amapá	16	0	0	0	-	0	0	0	0	-
Acre	22	1	4,5	0	-	0	0	0	0	-
Amazonas	62	2	3,2	1	1,6	120	118	98,3	118	98,3
Rondônia	52	1	1,9	0	0	0	0	0	0	-
Pará	144	3	2,1	2	1,4	350	242	69,1	242	69,1
Tocantins	139	3	2,2	1	0,7	142	138	97,2	128	90,1
Nordeste	1.794	352	19,6	223	12,4	15.663	11.160	71,3	10.505	67,1
Maranhão	217	10	4,6	6	2,8	439	35	8	34	7,7
Piauí	224	23	10,3	5	2,2	540	101	18,7	101	18,7
Ceará	184	175	95,1	157	85,3	10.690	8.237	77,1	7.702	72,0
Rio Grande do Norte	167	42	25,1	16	9,6	1.412	956	67,7	919	65,1
Paraíba	223	4	1,8	0	-	0	0		0	-
Pernambuco	185	9	4,9	2	1,1	46	28	60,9	19	41,3
Alagoas	102	7	6,9	1	1,0	15	15	100	12	80,0
Sergipe	75	64	85,3	28	37,3	2.114	1.595	75,4	1.540	72,8
Bahia	417	18	4,3	8	1,9	407	193	47,4	178	43,7
Centro-Oeste	466	55	11,8	30	6,4	4.020	2.749	68,4	2.617	65,1
Goiás	246	35	14,2	19	7,7	1.756	1.348	76,8	1.297	73,9
Mato Grosso do Sul	79	10	12,7	5	6,4	1.086	305	28,1	302	27,8
Mato Grosso	141	9	6,4	5	3,5	1.013	963	95,1	885	87,4
Distrito Federal^e^	1	1	-	1	100,0	165	133	80,6	133	80,6
Sudeste	1.668	668	40	601	36,0	31.881	29.880	93,7	27.824	87,3
Minas Gerais	853	57	6,7	31	3,6	3.420	3.158	92,3	3.054	89,3
Espírito Santo	78	17	21,8	12	15,4	1.178	1.178	100	1.090	92,5
São Paulo	645	586	90,9	552	85,6	24.642	24.193	98,2	22.374	90,8
Rio de Janeiro	92	8	8,7	6	6,5	2.641	1.351	51,2	1.306	49,5
Sul	1.191	1.089	91,4	997	83,7	78.671	75.927	96,5	71.415	90,8
Paraná	399	344	86,2	304	76,2	23.521	21.118	89,8	20.212	85,9
Santa Catarina	295	260	88,1	234	79,3	10.495	10.236	97,5	9.682	92,3
Rio Grande do Sul	497	485	97,6	459	92,4	44.655	44.573	99,8	41.521	93,0
BRASIL	5.570	2.174	39,0	1855	33,3	130.847	120.214	91,9	112.849	86,2

Fonte: Sisagua.

^a^ Total de municípios segundo o IBGE.

^b^ Filtro 1: Número de municípios com 4 meses ou mais de informação.

^c^ Filtro 2: Número de registros após a exclusão de valores “0,000”.

^d^ Filtro 3: Número de registros após a exclusão de valores aberrantes na distribuição por município.

^e^ Dados de Brasília referem-se ao Plano Piloto, excluindo-se, portanto, as demais regiões administrativas.

No que se refere aos registros, 58,6% deles apresentaram valores dentro do intervalo de concentração considerado ótimo para a prevenção da cárie (0,445–0,944 mgF/L), 33,6% dos valores estavam abaixo desse intervalo (0,001–0,444 mgF/L), 6,5% eram valores altos (0,945–1,444 mgF/L) e 1,3% valores muito alto (> 1,444 mgF/L). A macrorregião Sudeste apresentou o maior percentual de registros no intervalo ótimo (88,2%), seguida pelas regiões Centro-Oeste (62,9%), Sul (48,7%), Nordeste (47,7%) e Norte (27,6%). Nos municípios com 50 mil ou mais habitantes, 74,4% dos registros estavam no intervalo ótimo, e, naqueles com menos de 10 mil habitantes, apenas 42,0% estavam nesse intervalo, uma diferença de 32,4 p.p. Em relação aos valores muito altos (> 1,444 mgF/L), que configuram uma água imprópria para o consumo humano, cabe destacar que quatro UF (Tocantins, Maranhão, Pernambuco e Goiás) apresentaram 10% ou mais de registros nessa situação (Tabela 2).

**Tabela 2:** Número e proporção dos valores de fluoreto na água segundo os intervalos de concentração, a macrorregião, a unidade da federação e o porte populacional. Brasil, 2015.

Macrorregião	Registros de concentração de fluoreto (mgF/L)^a^								Total de registros

	Baixa (0,001–0,444)		Ótimo (0,445–0,944)		Alta (0,945–1,444)		Muito Alta (> 1,444)		
			
	n	%	n	%	n	%	n	%	
Norte	293	60,0	134	27,5	47	9,6	14	2,9	488
Roraima	0	-	0	-	0	-	0	-	0
Amapá	0	-	0	-	0	-	0	-	0
Acre	0	-	0	-	0	-	0	-	0
Amazonas	39	33,1	79	66,9	0	0,0	0	0,0	118
Rondônia	0	-	0	-	0	-	0	-	0
Pará	242	100,0	0	0,0	0	0,0	0	0,0	242
Tocantins	12	9,4	55	43,0	47	36,7	14	10,9	128
Nordeste	3.914	37,3	5.008	47,7	1.370	13,0	213	2,0	10.505
Maranhão	18	52,9	12	35,3	0	0,0	4	11,8	34
Piauí	75	74,3	26	25,7	0	0,0	0	0,0	101
Ceará	2.184	28,4	4.190	54,4	1.175	15,3	153	1,9	7.702
Rio Grande do Norte	750	81,6	159	17,3	9	1,0	1	0,1	919
Paraíba	0	-	0	-	0	-	0	-	0
Pernambuco	0	0,0	0	0,0	0	0,0	19	100,0	19
Alagoas	0	0,0	10	83,3	2	16,7	0	0,0	12
Sergipe	859	55,8	528	34,3	134	8,7	19	1,2	1.540
Bahia	28	15,7	83	46,6	50	28,1	17	9,6	178
Centro-Oeste	482	18,4	1.646	62,9	124	4,7	365	14,0	2.617
Goiás	312	24,0	638	49,2	27	2,1	320	24,7	1.297
Mato Grosso do Sul	73	24,2	171	56,6	55	18,2	3	1,0	302
Mato Grosso	62	7,0	776	87,7	9	1,0	38	4,3	885
Distrito Federal^b^	35	26,3	61	45,9	33	24,8	4	3,0	133
Sudeste	2.348	8,5	24.548	88,2	752	2,7	176	0,6	27.824
Minas Gerais	463	15,2	2.344	76,8	151	4,9	96	3,1	3054
Espírito Santo	7	0,6	1081	99,2	2	0,2	0	0,0	1090
São Paulo	1.512	6,8	20.285	90,7	501	2,2	76	0,3	22.374
Rio de Janeiro	366	28,0	838	64,2	98	7,5	4	0,3	1.306
Sul	30.883	43,2	34.732	48,7	5.066	7,1	734	1,0	71.415
Paraná	4.615	22,8	14.060	69,6	1.490	7,4	47	0,2	20.212
Santa Catarina	1.430	14,8	5.173	53,4	2.676	27,6	403	4,2	9.682
Rio Grande do Sul	24.838	59,8	15.499	37,3	900	2,2	284	0,7	41.521
Porte populacional									
Menor que 10 mil	18.017	50,7	14.948	42,0	2.079	5,8	527	1,5	35.571
10 mil a < 50 mil	12.331	34,6	20.164	56,5	2.535	7,1	632	1,8	35.662
Maior ou igual a 50 mil	7.572	18,2	30.956	74,4	2.745	6,6	343	0,8	41.616
Brasil	37.920	33,6	66.068	58,6	7.359	6,5	1502	1,3	112.849

Fonte: Sisagua.

^a^ Resultados após a aplicação dos Filtros 1, 2 e 3.

^b^ Dados de Brasília referem-se ao Plano Piloto, excluindo-se, portanto, as demais regiões administrativas.

A Tabela 3 apresenta a sumarização dessas informações por número e percentual dos municípios, segundo UF, macrorregião e porte populacional. Depois da aplicação dos Filtros 2 e 3, 1.810 (32,5%) municípios apresentaram informação válida para o parâmetro fluoreto, dos quais 726 (40,1%) mostraram 80% ou mais dos registros dentro do intervalo de concentração ótima (0,445–0,944 mgF/L). Diferenças importantes foram observadas entre as regiões e dentro de cada macrorregião. A porcentagem de municípios com informação válida foi 83,6% na macrorregião Sul, com um padrão similar entre os seus estados. Nas demais macrorregiões os valores foram 0,7% (Norte), 4,7% (Centro-Oeste), 10,8% (Nordeste) e 35,7% (Sudeste). Os estados Sergipe, Ceará, São Paulo e o Distrito Federal se destacaram com valores mais altos nas suas respectivas regiões. Nas regiões com maior porcentagem de municípios com informação válida, a qualidade da fluoretação medida pela porcentagem de municípios que apresentavam 80% ou mais dos registros dentro do intervalo de concentração ótima foi distinta. Enquanto na macrorregião Sul esse percentual foi 20,9%, na macrorregião Sudeste esse valor foi 80,4%. Os valores decresceram conforme diminuiu o porte populacional: 54,3% entre aqueles com 50 mil ou mais habitantes; 41,3% entre aqueles com 10 mil ou menos de 50 mil habitantes; e 34,2% na categoria com menos de 10 mil habitantes. Considerando a taxa de conformidade sobre o total de municípios, esse padrão se alterou em relação aos Estados e às macrorregiões. Segundo o porte populacional, a taxa de conformidade foi praticamente igual na comparação dos municípios de 10 a 50 mil habitantes com aqueles de menos de 10 mil habitantes.

**Tabela 3:** Número e percentual de municípios com informações válidas e com muito boa qualidade da fluoretação segundo a macrorregião, a unidade da federação e o porte populacional. Brasil, 2015.

	Municípios					

	Total^a^	Com informações válidas^b^		Taxa de conformidade		
		
	n	n	%	n	%^c^	%^d^
Norte	450	3	0,7	0	-	-
Roraima	15	0	-	0	-	-
Amapá	16	0	-	0	-	-
Acre	22	0	-	0	-	-
Amazonas	62	1	1,6	0	-	-
Rondônia	52	0	-	0	-	-
Pará	144	1	0,7	0	-	-
Tocantins	139	1	0,7	0	-	-
Nordeste	1.794	193	10,8	32	16,6	1,8
Maranhão	217	3	1,4	0	-	-
Piauí	224	1	0,5	0	-	-
Ceará	184	137	74,5	28	20,4	15,2
Rio Grande do Norte	167	16	9,6	0	-	-
Paraíba	223	0	-	0	-	-
Pernambuco	185	2	1,1	0	-	-
Alagoas	102	1	1,0	1	100,0	1,0
Sergipe	75	27	36,0	1	3,7	1,3
Bahia	417	6	1,4	2	33,3	0,5
Centro-Oeste	466	22	4,7	7	31,8	1,5
Goiás	246	16	6,5	5	31,3	2,0
Mato Grosso do Sul	79	2	2,5	0	-	-
Mato Grosso	141	3	2,1	2	66,7	1,4
Distrito Federal	1	1	100,0	0	-	0,0
Sudeste	1.668	596	35,7	479	80,4	28,7
Minas Gerais	853	26	3,1	15	57,7	1,8
Espírito Santo	78	12	15,4	12	100,0	15,4
São Paulo	645	552	85,6	452	81,9	70,1
Rio de Janeiro	92	6	6,5	0	-	-
Sul	1.191	996	83,6	208	20,9	17,5
Paraná	399	304	76,2	131	43,1	32,8
Santa Catarina	295	233	79,0	35	15,0	11,9
Rio Grande do Sul	497	459	92,4	42	9,2	8,5
Porte populacional						
Menor que 10 mil	2.451	833	34,0	285	34,2	11,6
10 mil a < 50 mil	2.464	688	27,9	284	41,3	11,5
Maior ou igual a 50 mil	655	289	44,1	157	54,3	24,0
Brasil	5.570	1.810	32,5	726	40,1	13,0

Fonte: Sisagua.

^a^ Total de municípios segundo o IBGE.

^b^ Municípios com informações válidas após aplicação dos Filtros 1, 2 e 3.

^c^ Porcentagem corresponde aos municípios que apresentaram 80% ou mais de registros dentro do intervalo de concentração ótima (0,445–0,944) em relação aos municípios que possuem informações válidas.

^d^ Porcentagem corresponde aos municípios que apresentaram 80% ou mais de registros dentro do intervalo de concentração ótima (0,445–0,944) em relação ao total de municípios.

Em 2015, 16 capitais e o Distrito Federal fluoretavam a água dos quais 11 (64,7%) tinham registros válidos de concentração do fluoreto e cinco (29,4%) mostraram 80% ou mais dos registros dentro do intervalo de concentração ótima.

## DISCUSSÃO

A principal contribuição deste estudo foi apresentar um script para a crítica de um *big data* com valores de concentração de fluoreto na água de abastecimento público, ilustrando, assim, sua aplicação para descrever os dados registrados em 2015 no Brasil. Os resultados mostraram variações importantes que exigem uma ação coordenada das diferentes esferas de governo responsáveis pela gestão do Vigiagua. A utilização da linguagem de programação do software R permitiu a organização e a análise de cerca de 134 mil registros do ano de 2015 distribuídos em diferentes municípios brasileiros sobre o parâmetro fluoreto presente em sistemas de abastecimento de água. A sumarização foi importante para identificar diferenças entre e dentro das regiões, tanto sobre a disponibilidade da informação sobre o parâmetro quanto sobre a qualidade da fluoretação da água de abastecimento público no Brasil.

O uso de softwares cada vez mais robustos exige equipamentos adequados para o funcionamento de programas e arquivos densos, somado aos custos para aquisição de planos que permitam o acesso ao recurso. A seleção das ferramentas de análise depende das metas do usuário, dos recursos mais utilizados no seu meio profissional e de soluções que sejam facilmente implementadas e favoreçam a tomada de decisão. De modo geral, deve-se buscar ferramentas flexíveis, de amplo uso, com boa documentação e robustez compatível com as metas pretendidas. Além da capacidade analítica ilimitada, o R é atualizado regularmente, apresenta ótimos recursos gráficos, pode ser utilizado de forma online e não tem custos para os usuários. Existem centenas de pacotes em vários servidores de universidades e institutos com funções, algoritmos e procedimentos para vários tipos de processamento de dados. Por ser adotado por uma grande comunidade de usuários, o R, em comparação com outras linguagens de programação, está menos propenso a erros. Visando a expansão do uso do R, pesquisas da área da saúde têm se dedicado a descrever o passo a passo das análises com essa linguagem, exemplificando funções para leitura e manipulação dos dados^14,17^, além de criar e disponibilizar pacotes para seu aperfeiçoamento^18^.

Entretanto, apesar de estar se tornando mais comum entre os pesquisadores, a sua utilização como uma ferramenta para gestão de dados ainda é limitada, uma vez que, requer o constante aperfeiçoamento de profissionais e a alfabetização em dados para o manejo de linguagens computacionais^19^. O script apresentado neste manuscrito pode ser adaptado a outros conjuntos de dados com algumas modificações, sendo uma alternativa flexível e livre. Dessa maneira, a alocação de recursos no orçamento dos órgãos de gestão em saúde e a oferta de atividades de capacitação são medidas importantes para superar as barreiras entre a tecnologia e os serviços de vigilância e de assistência à saúde.

Aproximadamente dois terços dos municípios brasileiros não possuíam informações válidas para o parâmetro fluoreto, a maior parte deles estava localizada nas regiões Norte, Nordeste e Centro-Oeste e tinha menos de 50 mil habitantes. Essa situação é praticamente igual à observada em 2008, quando pesquisadores constataram subalimentação e ausência dos dados sobre o fluoreto em 62,7% dos municípios brasileiros, principalmente em locais com piores indicadores socioeconômicos e sanitários. Eles alertavam para problemas na estrutura do Sisagua e em seu uso pelos municípios, recomendando alterações no sistema que visavam o aperfeiçoamento e o cumprimento da finalidade^9^.

No que pese o sistema de informação ter sido objeto de alterações muito importantes^8^, pode-se inferir que, decorridos sete anos, a implementação do programa nacional de vigilância da água em relação ao parâmetro fluoreto, após experimentar um estágio inicial de expansão até 2008, encontra-se praticamente estagnada. Consideramos essa situação como preocupante e que exige providências das autoridades sanitárias do país, entre outros órgãos, como as promotorias de justiça^20,21^ e de proteção e defesa do consumidor^22^. Em algumas UF, onde é baixa a frequência de informações válidas, a formulação de estratégias para inserir o tema na agenda dos gestores da saúde é urgente.

Em 2005, das 17 capitais brasileiras que eram fluoretadas, incluindo o DF, apenas 29,4% realizavam as etapas de coleta, análise e divulgação do parâmetro fluoreto^23^. Passados dez anos, a proporção subiu para 64,7%. Estudo multicêntrico em municípios brasileiros com mais de 50 mil habitantes indicou que 2/3 deles eram providos pela fluoretação da água, em que 53% realizavam a vigilância da concentração do fluoreto com base em dados de heterocontrole, com maiores porcentagens para as regiões Sul e Sudeste^15^. Segundo esse estudo, 44,1% dos municípios com mais de 50 mil habitantes tinham informações válidas a partir de dados de heterocontrole. A diferença pode estar ligada ao desenho de cada estudo. Enquanto naquele estudo a estimativa foi calculada para o período entre 2010 e 2015, e incluiu apenas os municípios fluoretados com cobertura populacional acima de 49,9%, neste a estimativa levou em consideração apenas o ano de 2015 e incluiu todos os municípios com frequência de informações válidas no sistema, independentemente da proporção da população coberta pela fluoretação.

A superação das limitações atuais é fundamental para o monitoramento e avaliação da exposição populacional ao fluoreto. A identificação das áreas onde é muito baixo o grau de implementação do programa de vigilância pode guiar a tomada de decisão. Os resultados alcançados com iniciativas para melhoria, tanto na cobertura quanto na qualidade da notificação do sistema de mortalidade pelas equipes de vigilância epidemiológica dos níveis federal, estadual e municipal, são reveladores da relevância dessas ações^24^, que devem assumir a forma de um esforço permanente a fim de reduzir as diferenças regionais na qualidade dos registros^25^.

A baixa qualidade da informação em muitos municípios pode estar relacionada às dificuldades relativas à disponibilidade de recursos estruturais necessários para assegurar a alimentação adequada do sistema, tais como, computadores apropriados e acesso à internet no ambiente de trabalho^26^. Além disso, os desafios da sua estruturação, como a referência no registro dos dados e na transparência na divulgação de informações sobre o abastecimento de água no Brasil, também incluem a conscientização dos sujeitos envolvidos sobre a necessidade de inserção de dados no sistema e sua importância para o gerenciamento dos riscos à saúde associados ao abastecimento de água no país^8^.

Em relação às informações válidas, mais da metade estavam dentro do intervalo de concentração ótimo. De modo similar às evidências sumarizadas por uma revisão da literatura^4^, as desconformidades foram proporcionalmente mais elevadas em direção a valores baixos, que poderiam significar aumento do risco de cárie, do que em direção a valores acima do intervalo de concentração ótima, que poderiam implicar em aumento do risco de fluorose dentária. Na Inglaterra, as concentrações de fluoreto em áreas providas pela tecnologia de saúde pública também foram mais baixas do que a meta fixada em 1 mgF/L^6^. Entre os fatores que poderiam ocasionar as oscilações de concentração, têm sido destacados a ausência de equipamento/substância fluoretante; infraestrutura laboratorial e técnica; a dificuldade técnico-operacional devido à falta de programas de treinamento; e a complexidade da rede de distribuição^4,6^. Entretanto, grandes disparidades foram observadas entre e dentro das regiões, podendo-se supor que o sistema de informação pode não estar sendo utilizado de forma oportuna pelo agente público para alertar os responsáveis pelo tratamento da água nos sistemas de abastecimento quanto às ações corretivas necessárias.

Dentre os municípios que possuíam informações válidas sobre o parâmetro fluoreto, menos da metade exibiram 80% ou mais registros no intervalo de concentração ótima. As maiores porcentagens foram observadas na macrorregião Sudeste e nos municípios com 50 mil ou mais habitantes. Estudo no estado de São Paulo mostrou que os municípios que não tinham atingido esse padrão possuíam menor porte populacional, menor renda *per capita* e o abastecimento não era provido pela empresa estadual ajustado por outros indicadores municipais^7^. Pesquisa conduzida em uma importante região metropolitana brasileira mostrou que a qualidade da fluoretação da água foi maior quanto mais alto o valor do índice de desenvolvimento humano, o porte populacional, a cobertura da escovação dental supervisionada e mais baixa a taxa de mortalidade infantil e a razão entre os procedimentos de extração dentária e o total de procedimentos básicos^27^.

Como a redução do viés socioeconômico na distribuição da cárie dentária, em decorrência do ajuste adequado da concentração do fluoreto na água, é assegurada quando a rede de abastecimento alcança bairros ricos e pobres^28^, uma hipótese para futuros estudos seria verificar se as condições socioeconômicas são piores entre os municípios brasileiros que não têm cumprido as obrigações atinentes à vigilância da água em relação ao parâmetro fluoreto. A falta de exposição populacional ao fluoreto na água no abastecimento dos territórios onde o ajuste da concentração poderia trazer mais benefícios em decorrência das dificuldades de acesso a outras fontes de fluoreto para proteção da dentição humana, configura uma fonte de injustiça social, podendo, ainda, interferir no custo-benefício dessa medida, uma vez que, além de aumentar o risco de cárie dentária, os gastos com a implantação e manutenção da fluoretação não estariam sendo acompanhados pelos benefícios esperados na saúde pública.

Uma das limitações deste estudo consiste na interpretação do alcance dos achados. Nesse sentido, é importante destacar que foram considerados todos os municípios com quatro ou mais meses de registro, e que o critério mais refinado, incluindo somente os municípios com seis ou oito meses de registro, poderia gerar resultados distintos. Contudo, o critério adotado possibilitou a comparação entre dados de sete anos atrás e teve como referência a heterogeneidade do processo de implementação do Vigiagua no país. Outro ponto a ressaltar trata-se da necessidade de adaptações no script original e a criação de novos filtros para a avaliação de outros parâmetros de qualidade da água, atendendo a normativa vigente. Além disso, o R disponibiliza diferentes ferramentas estatísticas que poderiam complementar a análise descrita, que, neste caso, foi priorizado o tratamento de dados de acordo com os objetivos propostos.

O exame da qualidade dos dados registrados para 2015 mostrou que dois terços dos municípios brasileiros não possuíam informações válidas para o parâmetro fluoreto, um quadro indicativo de que a implementação do programa nacional de vigilância da água em relação ao parâmetro fluoreto não melhorou desde 2008. Entre os municípios que realizaram a observação direta de amostras de água provenientes da rede de distribuição, 40,1% deles mostraram um padrão de qualidade de fluoretação muito bom, com diferenças importantes por porte populacional, por macrorregião e pelas UF. O script para crítica e manuseio dos dados com recursos de programação do software R se mostrou bastante útil para a produção de informação voltada a tomada de decisão ancorada em método padronizado.

## Footnotes

a Material suplementar disponível em: http://www.cecol.fsp.usp.br/dcms/uploads/arquivos/1646748853_Material%20Suplementar%20RSP%202022%20vol%2022%20Paulino%20et%20al..pdf
